# Progressive Demyelination in the Presence of Serum Myelin Oligodendrocyte Glycoprotein-IgG: A Case Report

**DOI:** 10.3389/fneur.2018.00340

**Published:** 2018-05-15

**Authors:** Sara Gil-Perotin, Jéssica Castillo-Villalba, Joan Carreres-Polo, Arantxa Navarré-Gimeno, Javier Mallada-Frechín, Francisco Pérez-Miralles, Francisco Gascón, Carmen Alcalá-Vicente, Laura Cubas-Nuñez, Bonaventura Casanova-Estruch

**Affiliations:** ^1^Multiple Sclerosis and Neural Regeneration Research Group, Hospital Universitari i Politècnic La Fe, València, Spain; ^2^Neuroimmmunology Unit, Hospital Universitari i Politècnic La Fe, València, Spain; ^3^Radiology Department, Hospital Universitari i Politècnic La Fe, Valencia, Spain; ^4^Neuroimmunology Unit, Hospital Clínic de València, Valencia, Spain; ^5^Hospital Universitari de Elda, Alicante, Spain

**Keywords:** recurrent inflammatory optic neuropathy, NMO, multiple sclerosis, progression, spinal cord, cell-based assay, myelin oligodendrocyte glycoprotein

## Abstract

The clinical diagnosis of patients with autoantibodies directed to conformational myelin oligodendrocyte glycoprotein MOG-IgG, can be challenging because of atypical clinical presentation. MOG-IgG seropositivity has been reported in several demyelinating diseases, including relapsing opticospinal syndromes [in the neuromyelitis optica spectrum disorders (NMOSD) and less frequently, in multiple sclerosis (MS)], but it has rarely been associated with the progressive course of disease. To contribute to the characterization of MOG-related demyelination, we describe the case of a patient with progressive demyelinating opticospinal disease, IgG-oligoclonal bands (OCB), and serum MOG-IgG.

## Summary

The clinical diagnosis of patients with autoantibodies directed to conformational myelin oligodendrocyte glycoprotein MOG-IgG, can be challenging because of atypical clinical presentation (1–3). MOG-IgG seropositivity has been reported in several demyelinating diseases, including relapsing opticospinal syndromes [in the neuromyelitis optica spectrum disorders (NMOSD) and less frequently, in multiple sclerosis (MS)], but it has rarely been associated with the progressive course of disease (4). To contribute to the characterization of MOG-related demyelination, we describe the case of a patient with progressive demyelinating opticospinal disease, IgG-oligoclonal bands (OCB), and serum MOG-IgG.

## Case Description

**Figure 1:**
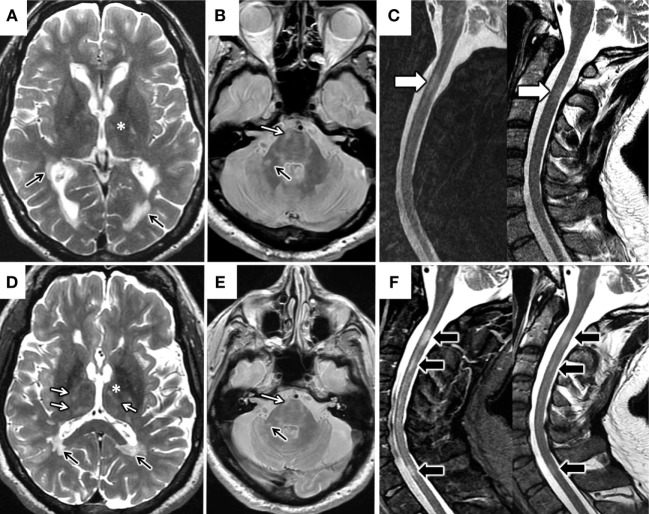
Changes in brain and spinal cord MRI. MRI 2010 **(A–C)**. Axial T2 FSE MRI image of the brain **(A)** demonstrates multiple periventricular lesions in the lateral ventricles (black arrows) and scarce thalamic lesions (*). Axial proton-density-weighted MRI image **(B)** shows predominant infratentorial lesions (white arrow in pons and black arrow in right middle cerebellar peduncle). Sagittal STIR and T2 FSE MRI images of the spinal cord **(C)** highlight the presence of a short segment spinal cord lesion (white arrow in C2). MRI 2016 **(D–F)**. Axial T2 FSE MRI image **(D)** shows increased in the number of thalamic lesions (* and white arrows) and stability of the periventricular lesions (black arrows). Axial proton-density-weighted MRI image **(E)** shows stability of the infratentorial lesions respect to 2010 (arrows). Sagittal STIR and T2 FSE MRI images of cervical spinal cord **(F)** demonstrate new short segment lesions affecting C3 and C7 levels respect (black arrows).

A white man of western European ancestry presented in 1997 with loss of visual acuity at age 26 although he did not seek medical assistance. He recovered *ad integrum*. Three years later, he experienced an episode of bilateral optic neuritis (ON). Symptoms went into complete remission after intravenous methylprednisolone. Since then, there was constant deterioration of gait without relapses despite intensive physical therapy and a diagnosis of a progressive phase of MS was made in 2003. We first attended the patient in 2010. He had received interferon and he was taking glatiramer acetate at that time. The gait problem persisted (he required two walking aids to walk more than 50 m) and was associated with mild dysphagia. Neurological examination showed dysarthria, central nystagmus, moderate dysmetria, symmetrical paraparesis with spasticity, and left facial and body hypoesthesia. He had preserved urine and bowel control. An expanded disability status scale (EDSS) score of 6.0 was calculated. Specific tests to exclude other causes were performed in our hospital despite the previous diagnosis of MS. An analysis of OCB in the cerebrospinal fluid (CSF) found IgG-OCB, but not IgM-OCB. Serum AQP4-IgG were not detected in serial samples. Brain and spinal cord MRI (FLAIR, TSE T_2_ sequence, PD, and T_1_ post gadolinium) was performed (Table 1; Figure 1). Brain MRI showed cortical, juxtacortical, and multiple periventricular lesions at the level of the lateral ventricles (Dawson’s fingers) with predominant infratentorial lesions (thalami, pons, and cerebellum). In the spinal cord, two nodular lesions (<2 vertebral segments) were observed at the level of C2–C3. There were no gadolinium-enhanced lesions (GEL). The patient fulfilled Barkhoff’s criteria, presenting with IgG-OCB, but not AQP4-IgG, and MS was considered the most likely diagnosis. Treatment with mitoxantrone [5 doses of 12 mg/m^2^ (20 mg)] had little effect on progression. In 2013, compassionate use of rituximab was initiated, although EDSS was 6.5 because of impairment of ataxia. Posterior MRI (in 2016) showed new lesions in the brain, cerebellum, brainstem, and spinal cord (in C4–C5, C5–C6, and C7) with cortical brain atrophy. T_2_-hyperintensity could be observed in the left corticospinal tract at the pons level as a sign of Wallerian degeneration. Despite signs of progressive demyelination, GELs were not detected.

**Table 1:** Number of lesions in T2-weighted MRI.

Date of the study	Brain					Diencephalus		Brainstem				Cerebellum	Spinal Cord	
	TV	LV	PV	JC	C	T	3V	Mid	Pons	Medulla	4V		Cervical	Dorsal
2010	2	>3	>3	>3	1	2	2	3	>3	1	1	>3	–	–
2012	–	–	–	–	–	–	–	–	–	–	–	–	>3 (<2 vb)	–
2016	2	>3	>3	>3	1	>3 ↑	2	3	>3=	1	1	>3=	>3 ↑ (<2 vb)	–

***TV**, temporal horn; **LV**, body of the lateral ventricle; **PV**, periventricular, Dawson’s fingers; **JC**, juxtacortical; **C**, cortical; **T**, thalamus; **3V**, third ventricle; **Mid**, midbrain; **4V**, fourth ventricle. **vb**, vertebral bodies*.

***↑** Increased number of lesions*.

**Figure 2:**
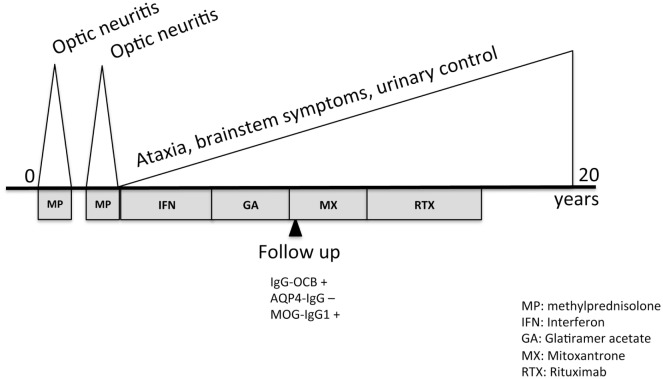
Timeline of clinical course and treatment.

At last visit, in 2017, his EDSS was 7.5, he was not capable of walking, had lost urine control, and had severe impairment of swallowing. Retrospectively, the analysis of MOG-IgG_1_ in the serum obtained in 2010 with a validated cell-based assay in our laboratory gave a positive result (titration 1:320) that was confirmed by an external center. We did not test CSF because the threshold of sensitivity of detection at titers under 1:640 (5). A diagram of the clinical course and administered treatments is displayed as Figure 2.

## Discussion

The identification of autoantibodies directed to conformational MOG (6, 7) in several demyelinating diseases (8) has encouraged clinicians to link their presence to patients with a characteristic disease pathogenesis or clinical course (9–12). MOG is expressed in the myelin sheath and the oligodendrocyte membrane and antibodies against MOG have been detected in the sera and CSF of patients with acute disseminated encephalomyelitis, NMOSD, relapsing isolated ON, and, more rarely, in MS. Technical limitations to detect the autoantibodies and borderline manifestations in seropositive patients with opticospinal disease has so far prevented the detailed characterization of MOG pathologies (3). However, recent multicentre studies with large numbers of MOG-IgG positive patients, have added more information regarding prognosis and disease course (13–15). The present case report describes a MOG-IgG-positive patient with progressive neurological deterioration after two episodes of ON. He was diagnosed as having progressive MS with brainstem predominance and myelitis, which evolved poorly despite aggressive immunosuppressive therapies. MOG-IgG-related entities are considered part of the NMOSD (16–19) and progressive deterioration has been described rarely (4, 13). Because of its atypical presentation, in this case it is interesting to analyze whether the assessed clinical progression was the result of ongoing deterioration secondary to inflammation, to a decrease in the reserve capacity of the brain upon formation of large pyramidal lesions (20), or both, or a deconditioning phenomenon. On the one hand, cumulative cortical atrophy was evident and new lesions in the spinal cord during MRI follow-up, without relapses, implied persistent disease activity. Moreover, the positivity for MOG-IgG in 2010, 13 years after the disease onset (in the absence of clinical attacks) supported the presence of active disease (1, 5, 21). On the other hand, GELs were not described in the MRI as hyperintensities in T_2_-weighted images in any of the studies, suggesting that chronic inflammation was not related to blood–brain barrier disruption. Left corticospinal tract degeneration in the pons could also have played a role in the progression of his disability, expressed as increasing spasticity, and loss of dexterity. However, there was no brainstem or myelitis clinical attack identified in the clinical course that could have caused an acute pyramidal lesion to blame. Instead, gait worsened progressively. Deconditioning, as an expression of deterioration because of lack of physical activity has been described in MS as related to chronic fatigue (22). In the present case, deconditioning was not a likely to be an influencing factor because of the intensive in-hospital physical therapy program and years of private physical therapy.

MRI findings with brainstem involvement shared some features with NMOSD and other cases of MOG-IgG (23). Our patient, in addition, presented with multiple brain lesions (periventricular, cortical, and juxtacortical) and short segment spinal cord lesions, more characteristic of MS. A high percentage of MOG-IgG-positive patients fulfilled criteria for both MS and NMOSD (13), but considering the latest McDonald criteria (24), that include IgG-OCB as a new criterion for dissemination in time, our patient fits better into the definition of MS. Supporting this, biopsy specimens from patients with MOG-IgG displayed features of pattern II MS with myelin injury (25–27) instead of the astrocytopathy that characterizes NMO. Nevertheless, it has yet to be elucidated whether there is enough data to define a separate entity distinct from MS or NMOSD. MOG-IgG positivity was initially shown to imply a benign prognosis (28). However, recent reports are consistent with more relapsing behavior and absence of response to certain therapies (13), mostly when MOG-IgG persist (1). In the case described in this report, corticoids served to recover visual acuity after ON, but afterward first-line therapies did not halt the clinical course and had to be switched to more aggressive treatments, including anti-CD20 therapies, with no effect on the progression of disability. We do not know whether anti-CD20 therapies had been used earlier, the disease course could have been slowed. Altogether, the present case emphasizes the need for MOG-IgG detection in atypical cases of MS (progressive or not) to design the therapeutic strategy and further investigation necessary to clarify the pathogenesis of MOG pathologies.

## Availability of Data and Supplementary Materials

Data available from the authors upon request.

## Ethics Statement

Written informed consent was obtained from the participant for participate in our study and for the publication of this case report in accordance with the Declaration of Helsinki and all research was conducted following legal and ethical requirements at the Research Institute of the Hospital La Fe and was approved by its Institutional Review Board.

## Author Contributions

BC-E and SG-P wrote the manuscript. SG-P, JC-V, and LC-N set up and performed cell-based assay. AN-G, JM-F, CA-V, FP-M, FG, and JC-P assisted the patient in the study. All authors made a critical revision of the manuscript.

## Conflict of Interest Statement

The authors declare that the research was conducted in the absence of any commercial or financial relationships that could be construed as a potential conflict of interest.
